# Validation of two-stage pancreatojejunostomy after pancreatoduodenectomy for patients with a soft pancreatic texture and assessment of the remnant pancreas texture before second surgery

**DOI:** 10.1016/j.sopen.2026.08.007

**Published:** 2026-08-30

**Authors:** Ryosuke Hatta, Yoichi Ishizaki, Jiro Yoshimoto, Kenji Takamori

**Affiliations:** a Institution for Environmental and Gender-Specific Medicine, Juntendo University, Chiba, Japan; b Department of General and Gastrointestinal Surgery, Juntendo University Urayasu Hospital, Chiba, Japan

**Keywords:** Pancreatoduodenectomy, Pancreatic Fistula, Two-stage pancreatojejunostomy, Pancreatic texture, CT attenuation

## Abstract

**Background:**

Two-stage pancreatojejunostomy (PJ) after pancreatoduodenectomy (PD) is one of the methods available for minimizing the possibility of grade C postoperative pancreatic fistula (POPF), especially in patients with a soft pancreatic consistency.

**Methods:**

We studied 131 patients with a soft pancreatic texture who underwent initial PD without PJ. The pancreatic tube was exteriorized through the abdominal wall incision. Three months later, we performed PJ, except for four patients died of recurrence. The remaining 127 patients underwent two-stage PJ. To assess the changes in pancreatic consistency after PD, computed tomography (CT) attenuation of the pancreas was measured before PD and compared with that after PD in 36 patients.

**Results:**

After initial PD, none of the patients suffered grade C POPF and postoperative mortality was zero. After the second operation, none of the patients suffered grade C POPF. One patient died due to recurrence of cancer. There was no mortality attributable to any adverse effect of POPF. The postoperative mortality rate was 0.7%. At the time of two-stage PJ, surgeons confirmed that texture of the remnant pancreas had changed from soft to hard. CT attenuation of the remnant pancreas before PJ was significantly lower than that of the pancreas body before PD (32.3 ± 10.0 vs. 40.5 ± 8.2, *p* < 0.001).

**Conclusion:**

Two-stage PJ can be considered to minimize likelihood of grade C POPF. The changes in CT attenuation before PD and before PJ provide objective confirmation of a change in pancreatic texture from soft to hard, thus largely accounting for the reduced incidence of fatal POPF.

## Introduction

Despite recent advances in surgical techniques and postoperative management, a significant proportion of patients undergoing pancreatoduodenectomy (PD) develop postoperative pancreatic fistula (POPF), which is the most frequent cause of postoperative death. Life-threatening POPF still occurs in 6.2–30.0% of such patients, with a mortality rate of 1.0–8.4% [1], [2], [3], [4], [5], [6].

The ultimate goal of pancreatic surgeons is to reduce to zero the incidence of in-hospital mortality after PD. An extensive range of methods for reducing the incidence of POPF have been suggested. These include technical adjustments such as modification of the pancreatojejunal anastomosis technique [7], reconstruction using pancreatogastrostomy [8], placement of pancreatic duct stents [9], perioperative infusion of somatostatin analogues [10], use of adhesive sealants [11], and early removal of external drainage [12]. Among pancreatic surgeons, it is accepted that in comparison with a hard pancreatic consistency, a soft pancreatic consistency is associated with a greater incidence of POPF after PD [10], [13], [14], [15], [16]. Two-stage pancreatojejunostomy (PJ) after PD is one of the methods available for minimizing the likelihood of adverse events related to pancreatic juice, especially in patients with a soft pancreatic consistency [17], [18]. In two-stage PJ, a pancreatostomy is created first with placement of an external tube, which is not passed through the jejunal loop, followed about 3 months later by second-stage reconstruction for PJ. Although this involves two surgical procedures, activation of the enzymes present in pancreatic juice and the critical adverse events associated with it can be prevented. Recently, we have adopted a two-stage PJ following PD for patients with a soft pancreatic consistency. The aim of the present study was to evaluate whether two-stage PJ after PD reduces total morbidity and the incidence of life-threatening POPF in patients with a soft pancreatic texture. Also, the difference between the pancreatic texture before and after initial PD was assessed in terms of CT-evident attenuation.

## Methods

Between January 2009 and April 2024 407 PDs were carried out at Juntendo University Hospital and Juntendo Urayasu Hospital. We adopted two-stage PJ for patients with a soft pancreatic texture, and 131 such patients underwent initial PD in which a PJ was not carried out. The pancreatic tube was exteriorized through the abdominal wall incision. About 3 months after the PD, we performed PJ, except in four patients who were unable to undergo the second operation due to rapid recurrence of their cancers. The remaining 127 patients underwent the two-stage PJ. This study was designed to review the 131 patients who underwent PD without PJ and the 127 patients who underwent two-stage PJ after PD. Informed consent was obtained from all individual participants included in the study. The study was approved by the ethics committee of Juntendo University, Urayasu Hospital (Approval No. E25–0052; Jun 2025).

### Surgical technique

#### Initial PD

All the patients underwent standard PD uniformly in accordance with the standardized Whipple procedures [19].

In brief:

1. To minimize intraoperative blood loss, early ligation of the inferior pancreatoduodenal artery was adopted for all patients [20]. Reconstruction was achieved according to the Child procedure.
2. An external tube pancreatojejunostomy was constructed using a polyvinyl chloride tube (Sumitomo Bakelite, Tokyo, Japan) without passing the tube through the jejunal loop. The size of the external pancreatostomy tube was set as the diameter of the main pancreatic duct. The cut plane of the pancreas was fixed to the jejunal wall with nonabsorbable sutures.
3. End-to-side choledochojejunostomy was performed 20 cm distally to the pancreatostomy, and end-to-side gastrojejunostomy was carried out 40 cm downstream. For decompression of the jejunal loop, a tube jejunostomy was created [21].
4. An omental flap was placed behind the pancreatojejunostomy to cover the stump of the gastroduodenal artery. Then, two drains were placed near the pancreatojejunostomy.

#### Two-stage PJ

About 3 months after the initial PD, we performed a PJ using the mucosal sutureless technique reported previously [22].

1. The pancreatostomy tube was exteriorized through the abdominal wall incision (Fig. 1a), and an abdominal midline incision was made along the previous scar.

**Fig. 1:**
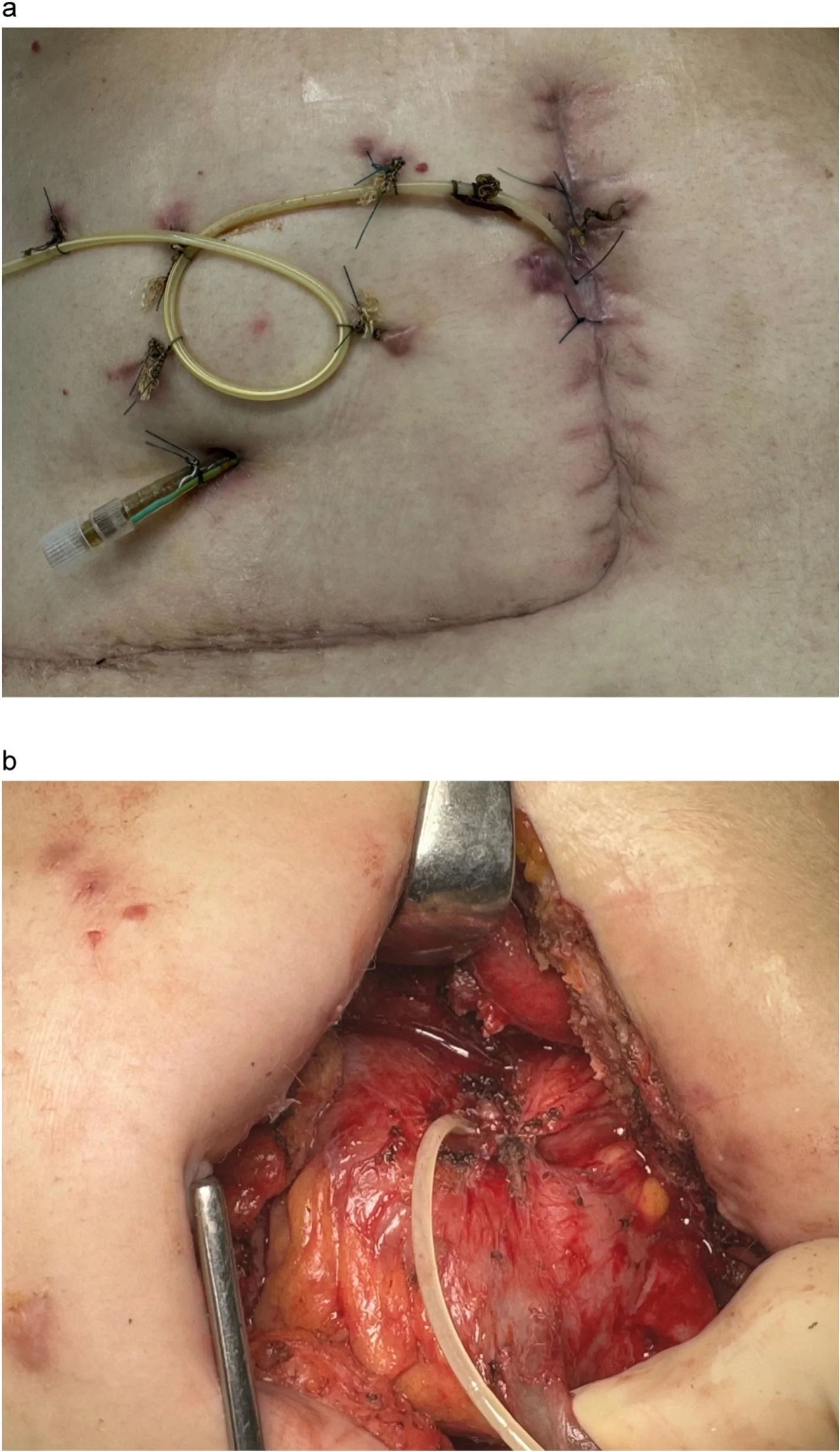

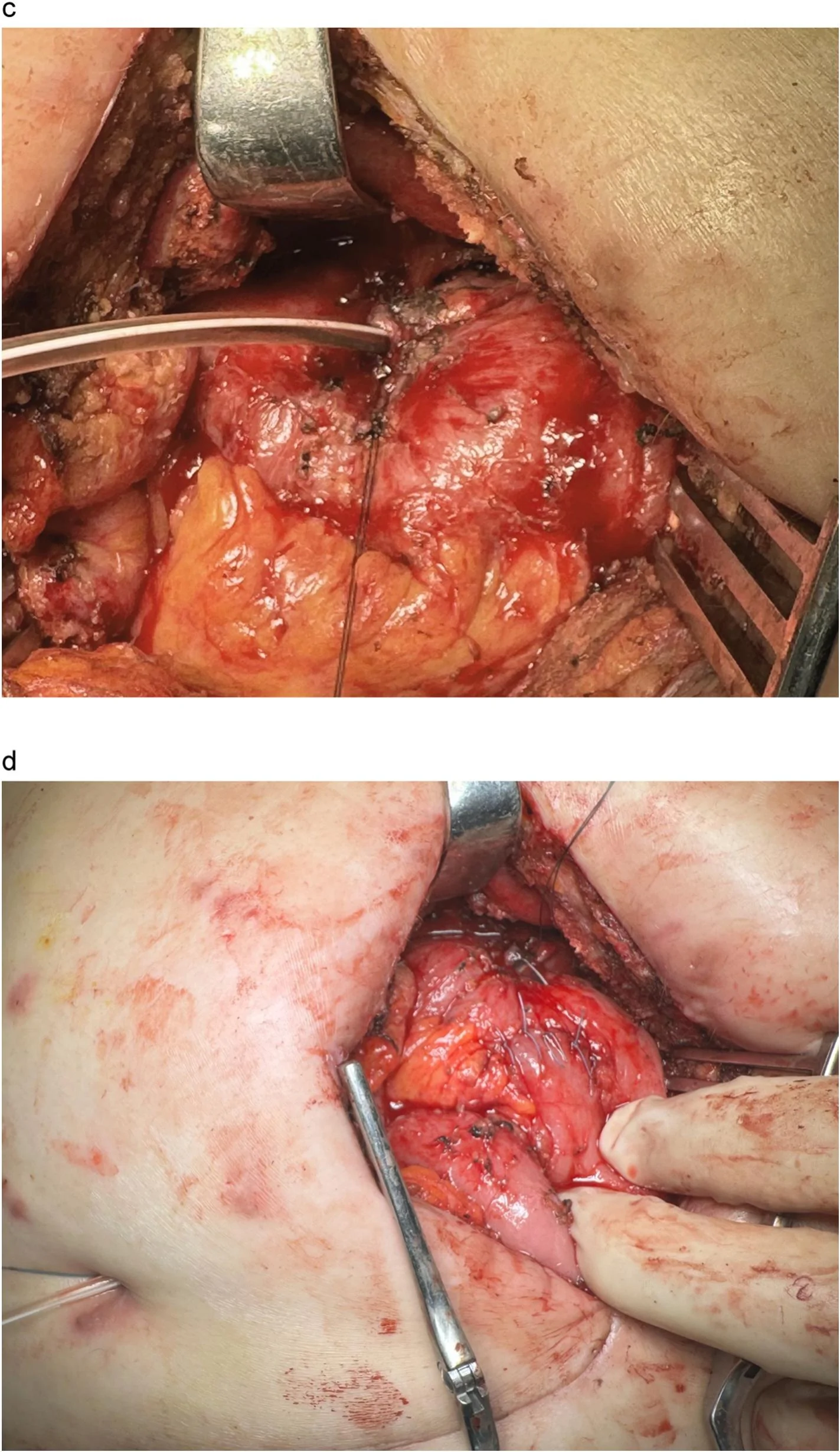

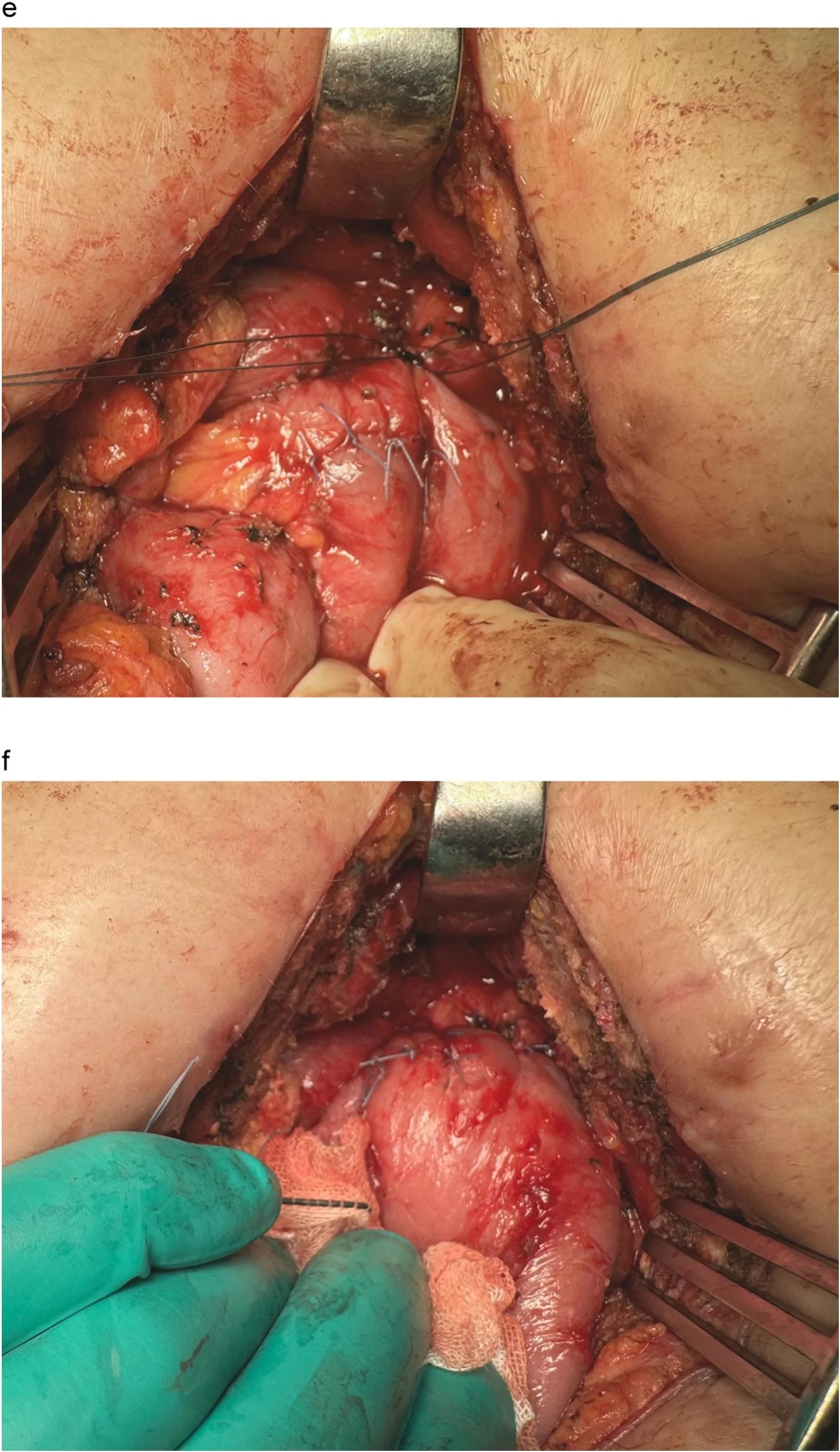
a The pancreatostomy tube was exteriorized through the abdominal wall incision. b Careful dissection around the external drainage tube was done and the point of tube insertion in the cut surface of the pancreas was exposed. c The tube was replaced by another that was one size thicker. d The stent tube was passed into the jejunal lumen and externalized through the jejunal loop. e The stent tube was ligated with two stay sutures to approximate the pancreatic ductal cut end and the tiny hole in the jejunum f Finally, a suture was added between the anterior parenchyma of the pancreas and the seromuscular layer of the jejunum.
2. We conducted careful dissection around the external drainage tube and exposed the point of insertion in the cut surface of the pancreas (Fig. 2b). The tube was replaced by another that was one size thicker (Fig. 1c).

**Fig. 2:**
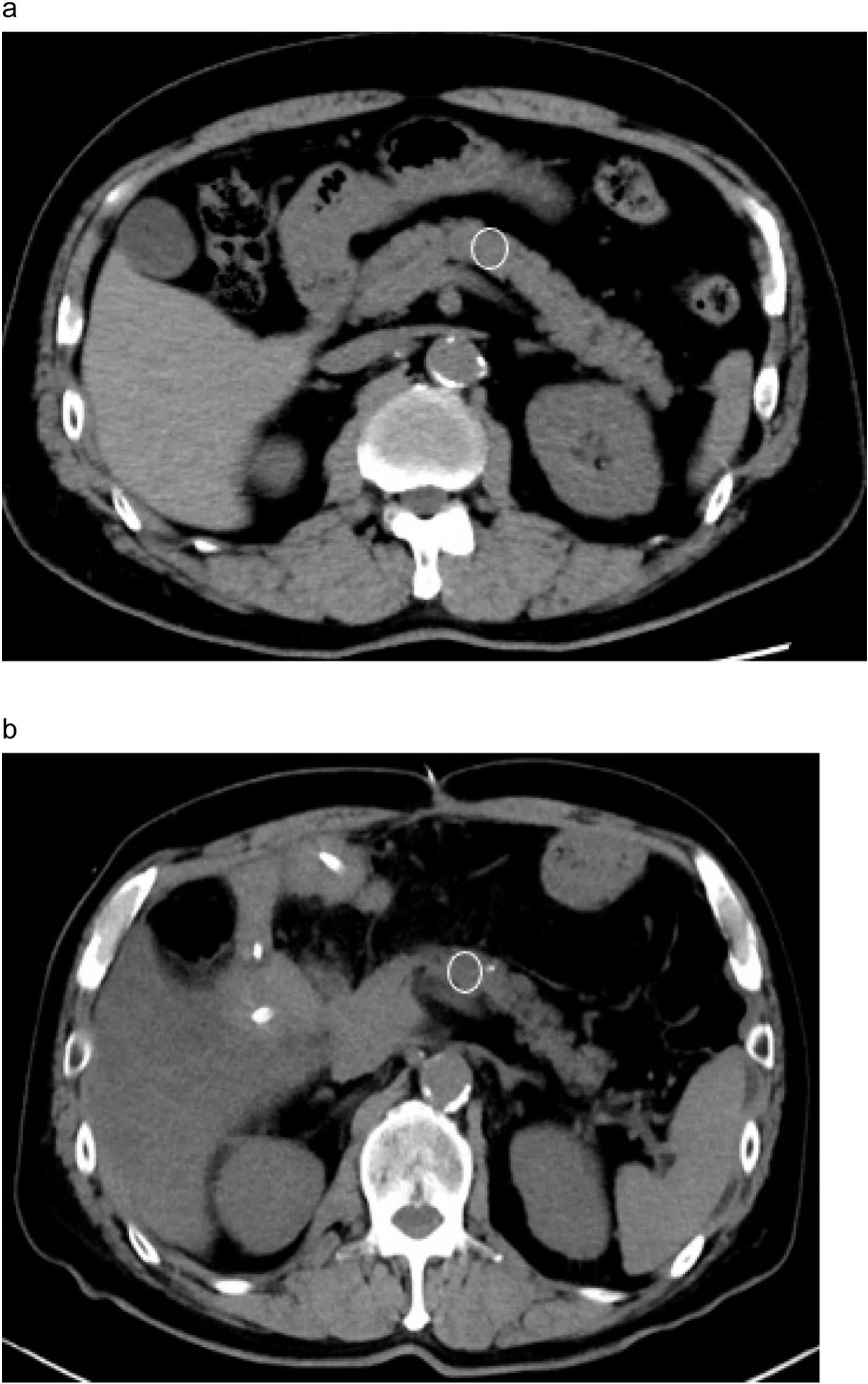
a Before initial PD, the CT attenuation value of the pancreas body was measured. Each region was a circular area 1.5 cm in diameter. b After initial PD, the CT attenuation values of the remnant pancreas was measured. Each region was a circular area 1.5 cm in diameter.
3. The stent tube was passed into the jejunal lumen and externalized through the jejunal loop (Fig. 1d). The jejunal side of the tube was held in place with a purse-string suture. The stent tube was ligated with two stay sutures to approximate the pancreatic ductal cut end and the tiny hole in the jejunum (Fig. 1e).
4. Finally, a suture was added between the anterior parenchyma of the pancreas and the seromuscular layer of the jejunum (Fig. 1f).

#### Evaluation of pancreatic consistency in terms of CT attenuation

To determine the effects of initial PD on the remnant pancreas, pancreatic computed tomography (CT) attenuation was evaluated after PD. Thirty-six patients who had undergone two-stage PD between July 2018 and April 2024 were enrolled. We retrospectively reviewed unenhanced CT images showing the CT attenuation of the pancreatic body one months before initial PD (Fig. 2a) and that in the same part of the remnant pancreas just before two-stage PJ (Fig. 2b).

#### Evaluation of the exocrine and endocrine functions of the remnant pancreas

We measured the serum albumin level as a substitute exocrine marker before and after PD in most recent 28 patients. Also, for the assessment of the endocrine function of the remnant pancreas, we measured and compared HbA1c levels before and after PD for the most recent 20 patients in whom diabetes mellitus was not a preoperative complication.

### Endpoint definitions

POPF was defined in accordance with the guidelines of the International Study Group on Pancreatic Fistula (ISGPF), i.e. an amylase-rich drainage fluid with an amylase concentration more than 3 times the serum concentration collected via the intraoperatively placed drain from day 3 onwards. POPF was graded according to its clinical impact on the patient's hospital course (grade BL, B, or C). Clinically relevant pancreatic fistulas were defined as ISGPS grade B or C. Life-threatening POPF was defined as ISGPS grade C. Grade C POPF is a type that requires reoperation or which can potentially lead to single or multiple organ failure [23]. Any types of relevant postoperative morbidity were recorded and classified as proposed by Clavien and co-workers. Severe morbidity was defined as a Clavien grade III or greater.

For each patient who underwent initial PD, the fistula risk score (FRS) was calculated. The FRS can be classified into four risk levels: (1) negligible risk (0 points); (2) low risk (1–2 points); (3) intermediate risk (3–6 points); (4) high risk (7–10 points) [24].

### Statistical analysis

Continuous variables were expressed as mean ± SD and statistical analysis of laboratory data was done using a paired samples *t-*test. Categorical variables were compared using the chi-squared test. Calculations were made using StatView computer software (SAS Institute Inc., SAS Campus drive, Cary, North Carolina). Differences at *P* < 0.05 were considered statistically significant.

## Results

Table 1 shows the perioperative data related to the initial PD. Indications for PD were pancreatic head cancer (*n* = 23), intraductal papillary mucinous neoplasms (IPMNs) (*n* = 9), pancreatic neuroendocrine tumor (p-NET) (*n* = 10), distal bile duct cancer (*n* = 47), ampullary cancer (*n* = 27), duodenal cancer (*n* = 4), and others (*n* = 11). In all patients, the consistency of the remnant pancreatic parenchyma was assessed as soft by the surgeons. The median size of the main pancreatic duct was 2.5 mm (1.3–4.5). POPF occurred in 42 patients, and was classified as grade B in all of them. None of the patients suffered grade C POPF. There were 141 complications in 106 patients. However, most events were classified as Grade I or II (97%). Only four patients developed Grade IIIa complications. Two patients developed complications associated with the externalized pancreatic tube. In one case, dislocation of the pancreatic tube occurred, and re-insertion was carried out under fluoroscopic guidance. In the other case, pancreatitis occurred due to tube obstruction. The obstruction was cleaned using a guidewire, and recanalization was achieved. No patients died of POPF or other complications. The 30-day postoperative mortality rate and the 90-day postoperative mortality rate were both zero.

**Table 1:** Perioperative data of the initial PD in the patients undergoing two-stage PJ (*n* = 131).

Age (years)	69 (36–86)
Gender(Male/Female)	78/53
Etiology	
Pancreas head cancer	23
IPMN	9
Pancreas neuroendocrine tumor	10
Bile duct cancer	47
Ampullary cancer	27
Duodenal cancer	4
Others	11
Operation time (min)	553 (331–897)
Intraoperative bleeding (mL)	240 (35–3450)
Need for red cell blood transfusion (Yes/No)	7/124
The size of the pancreatic duct (mm)	2.5 (1.3–4.5)
Texture of the remnant pancreas (soft/hard)	131/0
Hospital stay (day)	40 (16–248)
POPF Grade BL/B/C	40/42/0
POPF Grade B	42 (32%)
POPF Grade C	0 (0%)
Total number of patients who developed postoperative complications	106 (81%)
Total Events	141
Grade I, II, IIIa	141 (100%)
Grade IIIb ≤	0 (0%)
Total number of the complication associated to the externalized pancreatic tube	2(1.5%)
Mortality according to POPF	0 (0%)
30-day mortality	0 (0%)
90-day mortality	0 (0%)

Table 2 shows the perioperative data related to the second operation in patients who underwent two-stage PJ. Four patients were unable to undergo the second operation because of rapid recurrence of the cancer, and 127 patients underwent the second operation after two-stage PJ. In all patients, the consistency of the remnant pancreatic parenchyma was assessed as hard by the surgeons. The median operation time was 144 min (range 68–491 min), and the median intraoperative blood loss was 20 mL (range 5–320 mL). None of the patients received red cell blood transfusion. The median length of hospital stay was 19 days (range 8–145 days).

**Table 2:** Perioperative data of the second operation in the patients undergoing two-stage PJ (*n* = 127).

Age (years)	69 (36–86)
Gender(Male/Female)	75/52
Duration between the two surgery (day)	99 (73–331)
Operation time (min)	144(68–491)
Intraoperative bleeding (mL)	20 (5–320)
Need for red cell blood transfusion (Yes/No)	0/127
Texture of the remnant pancreas (soft/hard)	0/127
Hospital stay (day)	19(8–145)
POPF Grade BL/B/C	24/19/0
POPF Grade BC	19 (15%)
POPF Grade C	0 (0%)
Total number of patients who developed postoperative complications	61 (48%)
Total Events	63
Grade I,II, IIIa	62 (98%)
Grade IIIb ≤	1 (2%)
Total number of the complication associated to the externalized pancreatic tube	1(0.8%)
Mortality according to POPF	0 (0%)
30-day mortality	1 (0.7%)
90-day mortality	1 (0.7%)

After the second operation, POPF occurred in 19 patients, and was classified as grade B in all of them. None of the patients suffered grade C POPF. Thirty-seven patients suffered 63 complications including POPF. Only one patient developed complications classified as grade IIIb. In that case, because of pancreatic duct stent dislodgement, reoperation was performed. No patients died of POPF. Only one patient died of recurrent pancreatic cancer on postoperative day 27. The 30-day postoperative mortality rate and the 90-day postoperative mortality rate were both 0.7%.

**Fig. 3:**
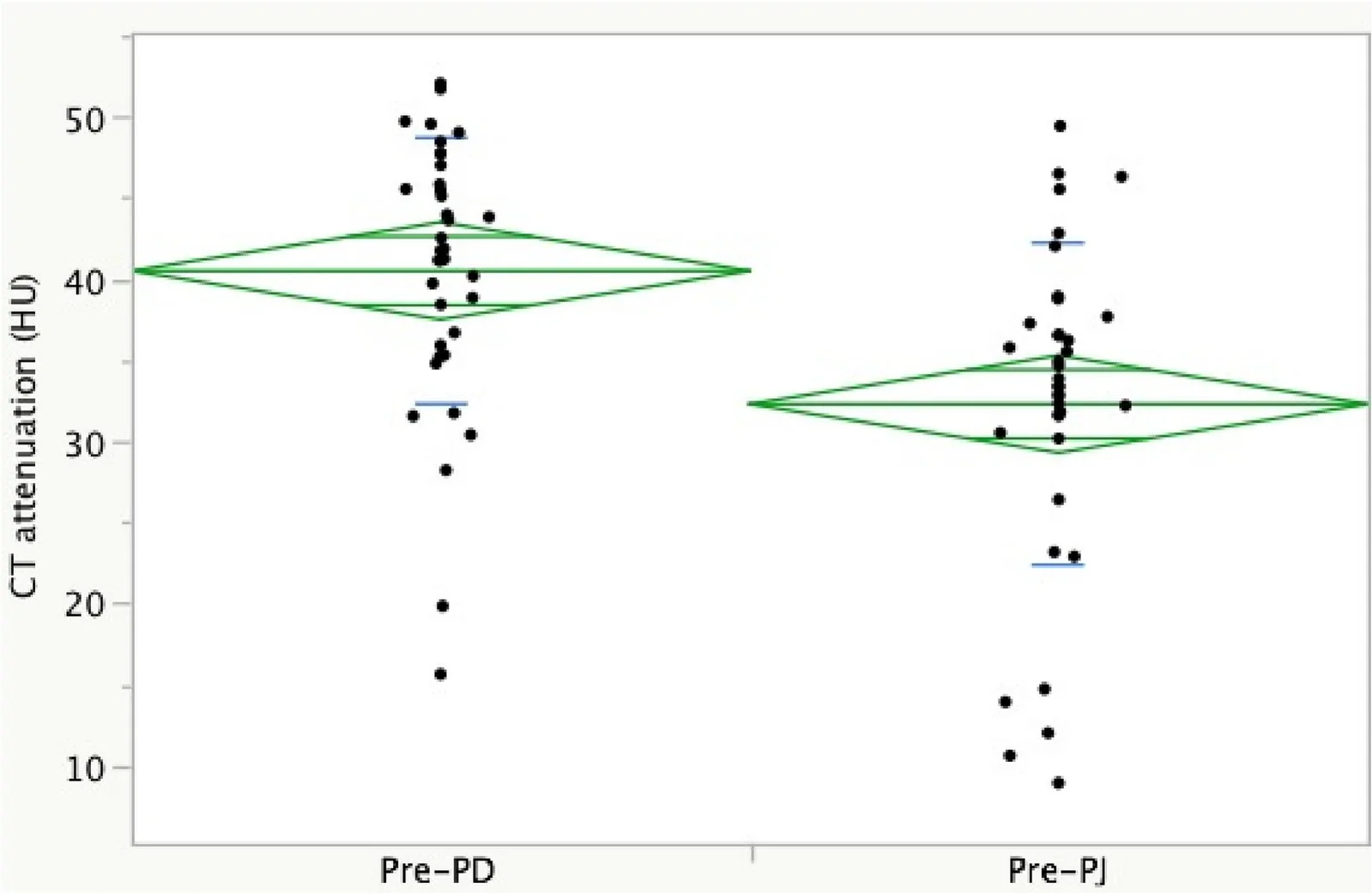
Comparison of pancreatic CT attenuation before initial PD with that after initial PD (*n* = 36).

Fig. 3 shows CT evaluation of the remnant pancreas. The mean remnant pancreatic CT attenuation before PD (41.8 ± 8.0) was significantly higher than that before two-stage PJ (33.6 ± 10.0) (*p* < 0.001, paired *t*-test).

**Fig. 4:**
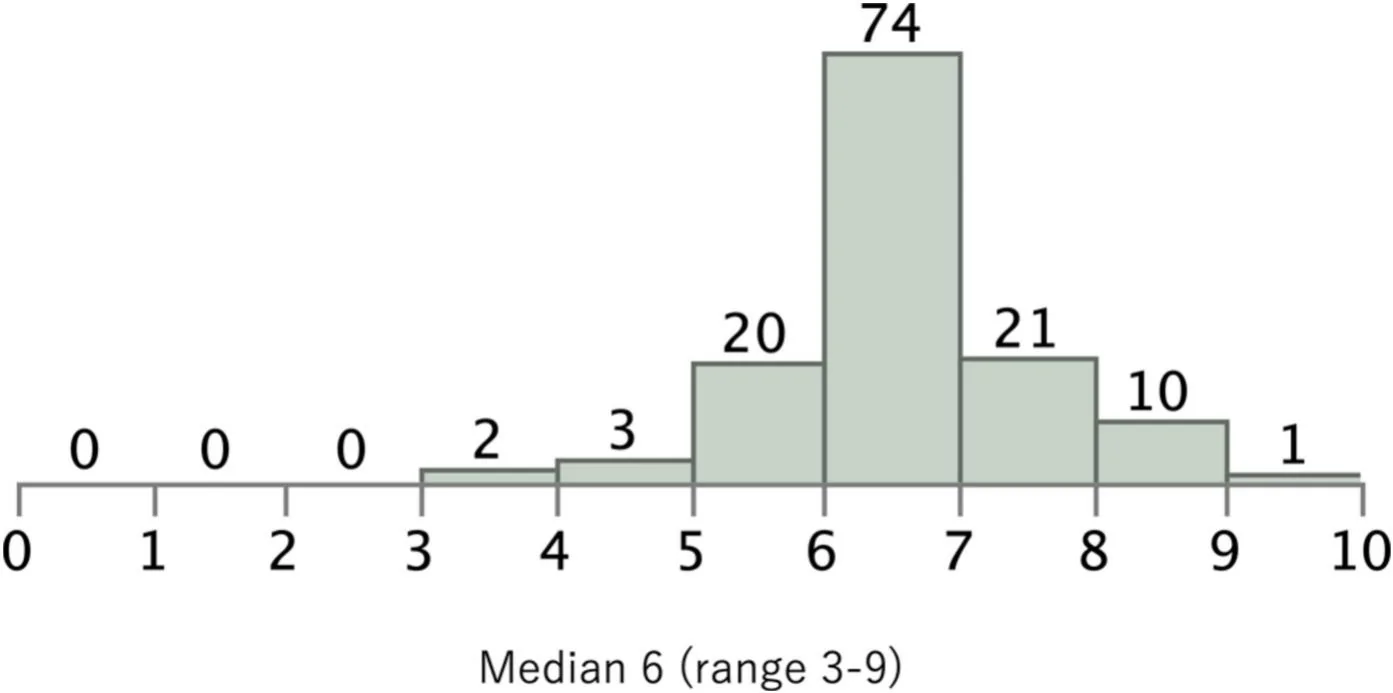
Among 131 patients who underwent initial PD, none were classified as negligible or low risk (0%), 99 (76%) were classified as intermediate risk, and 32 (24%) were classified as high risk.

Among 131 patients who underwent initial PD, none were classified as negligible or low risk (0%), 99 (76%) were classified as intermediate risk, and 32 (24%) were classified as high risk (Fig. 4).

Table 3 shows the serum albumin level and HbA1c level before and after PD.

**Table. 3:** Changes in serum albumin levels and HbA1c levels before and after PD.

	Before PD	1 year after PD		2 years after PD		3 years after PD	
Alb (mg/dL)	3.8 ± 0.4 (*n* = 28)	3.9 ± 0.4 (*n* = 25)	*P* = 0.59	3.8 ± 0.6 (*n* = 18)	*p* = 0.34	3.9 ± 0.3 (*n* = 15)	*p* = 0.39
HbA1c (%)	5.8 ± 0.6 (*n* = 20)	5.9 ± 0.6 (*n* = 16)	*P* = 0.40	5.8 ± 0.9 (*n* = 11)	*P* = 0.58	6.0 ± 0.9 (*n* = 13)	*P* = 0.93

Albumin returned to the preoperative level by 1 year after PD and remained almost constant until 3 years after PD. In two patients who showed new onsets of DM after PD, the average HbA1c levels after 1, 2 and 3 years PD did not differ from those before PD.

## Discussion

Since mortality after PD largely results from POPF grade C and intra-abdominal hemorrhage from ruptured aneurysms [25], the key to excellent outcomes after PD is undoubtedly reduction in the incidence of these complications. In comparison with a hard pancreatic consistency, a soft pancreatic consistency is associated with a higher incidence of POPF. There are three possible reasons why patients with a soft, fragile pancreas could be considered at high risk for POPF [10]. First, most soft pancreases have a small duct, for which secure duct-to-mucosa PJ is difficult. Second, a soft pancreas is more easily injured directly or via ischemia by stitches placed between the pancreatic cut end and the seromuscular layer of the digestive tract. Third, a soft pancreas has good exocrine function, secreting a higher volume of pancreatic juice rich in proteolytic enzymes.

A soft pancreas is not the only risk factor for POPF after PD. An extensive analysis of pre- and interoperative variables revealed three other distinct factors: Pancreatic duct diameter less than 3 mm; ampullary, duodenal, cystic, or islet cell pathology; and excessive intraoperative blood loss. The FRS is calculated on the basis of these four factors. POPF rates increased significantly across FRS risk levels: negligible, 0%; low, 4.9%; moderate, 20.5%, and high, 30.4% [26]. Based on the FRS assessment, all patients included in the present study were classified as having an intermediate or high risk of POPF. However, none of the patients died of POPF.

In PD without PJ, pancreatic secretion from the main pancreatic duct can be completely drained outside the abdominal cavity through the drainage tube inserted in the main pancreatic duct. Therefore, this technique can overcome the above three problems. Moreover, although extravasation of pancreatic secretions from the pancreatic stump still remains, the pancreatic juice is not activated by enteric contents and/or bile juice. After initial PD all patients were able to survive and be discharged from hospital.

While 19 of the 127 patients undergoing two-stage PJ suffered grade B POPF, none of them developed grade C POPF. Only one patient who underwent two-stage PJ developed a Grade IIIb complication. This patient was found to have a dislodged pancreatic duct stent after pancreatic jejunal anastomosis and underwent reoperation, but did not develop POPF and had a good postoperative course. One patient died of recurrence of pancreatic cancer on postoperative day 27, but this was unrelated to the PF.

Most surgeons can predict whether a second operation will be difficult if there is severe adhesion around the cut surface of the remnant pancreas. However, we found that the adhesion was not so severe, and we were easily able to find the point of insertion of the stent tube and replace it with a new one. The median operation time for two-stage PJ was 144 min, and the median intraoperative bleeding volume was 20 mL.

Two-stage PJ is not technically demanding, but one drawback of this approach is that the stent tube may become obstructed. If such obstruction occurs, acute pancreatitis is inevitable. The surgeon must therefore instruct patients to measure the volume of their secreted pancreatic juice every day. If the volume decreases, the patient should visit the hospital, and the pancreatic tube can then be washed out with normal saline.

In the present study, although the pancreatic texture was soft at the time of initial PD, the consistency of the remnant pancreatic parenchyma at the time of the second surgery was assessed as hard by the surgeons in all patients. CT attenuation of the remnant pancreas was significantly lower than that of the pancreas body before PD. Sano et al. reported that CT attenuation of the pancreas was inversely correlated with the grade of fibrosis. The fibrosis grade was significantly higher for a hard pancreas than for a soft pancreas. They concluded that CT attenuation might be helpful for preoperatively prediction of POPF development after PD [27]. On the other hand, Yong et al. reported that a CT attenuation of <40 was a risk factor for pancreatic fistula and that CT values were negatively correlated with fistula severity [28]. CT values in the present cohort tended to be lower after PD, which seems to contradict their reports, but might have reflected the change in the nature of the residual pancreas occurring after PD. This is the first reported study to have demonstrated a change in the texture of the pancreas from soft to hard three months after the complete exteriorization of the pancreatic tube.

Delayed reconstruction strategy may induce fibrosis or parenchymal atrophy, potentially compromising exocrine and endocrine function of the remnant pancreas. Several pancreatic exocrine function tests have been reported. Although the secretin-cholecystokinin test precisely evaluates pancreatic enzyme output, we did not apply such a direct function test for the patients who underwent PD. Therefore, we measured the serum albumin level as a substitute exocrine marker, and the HbA1c level was assessed for evaluation of endocrine function. In the present series, most of the patients who underwent two-stage PJ did not exhibit significant prolonged exocrine or endocrine dysfunctions.

One limitation of the present study was the lack of a control group of patients with a soft pancreas who underwent one-stage PD. Although postoperative mortality has decreased to <5% within the last decades due to technical and medical advances, POPF grade C after PD remains a serious complication with a high level of morbidity and mortality. As our goal is to reduce the mortality rate to zero. we believe that all patients with a soft pancreas should be treated by two-stage PD.

The recent trend for early removal of intraperitoneal drains following PD may represent a key factor for improving the outcome of “fast-track” protocols and reducing the period of hospitalization, thus contributing to high-quality, cost-effective care. In Japan, total hospitalization costs are not so high because the national insurance system covers most of the expense. For this reason, postoperative stay is not considered to be a critical factor, and continuous placement of open drains cranially and caudally to the pancreatojejunostomy is often the policy. These are removed at the surgeon's discretion after the patient has resumed a regular diet and when the possibility of pancreatic fistula can be excluded. This would account for the long hospital stay after initial PD.

Over the past decade, accumulation of high-level evidence has established that minimally invasive pancreatic resection is a safe and effective alternative to open surgery when performed by experienced teams. In our institution minimally invasive distal pancreatectomy for patients with low-grade pathological diseases such as IPMN or neuroendocrine tumor has been introduced. However, owing to the inherent technical challenges associated with PD, including complex anastomoses and extended operation time, we have not yet begun to adopt it. To ensure safe implementation of minimally invasive PD as an initial procedure, we are now expanding out dedicated training, simulation-based education, and institutional support.

## Conclusion

Although two operative procedures are necessary, a two-stage PJ should be considered for patients with a soft pancreatic consistency in order to minimize the likelihood of complications that would be worse than grade III and grade C POPF. Furthermore, the changes in CT attenuation values before and after PD may reflect a change in pancreatic consistency from soft to hard, suggesting that such a change may help to reduce the likelihood of fatal pancreatic fistula.

## CRediT authorship contribution statement

**Ryosuke Hatta:** Writing – original draft, Resources, Investigation, Formal analysis, Data curation. **Yoichi Ishizaki:** Writing – review & editing, Writing – original draft, Validation, Supervision, Resources, Methodology, Investigation, Data curation, Conceptualization. **Jiro Yoshimoto:** Resources, Investigation, Data curation. **Kenji Takamori:** Supervision.

## Ethics approval

Ethics approval was granted by the ethics committee of Juntendo University, Urayasu Hospital (Approval No. E25–0052; Jun 2025).

## Funding

This research received no external funding.

## Declaration of competing interest

The authors report no proprietary or commercial interest in any product mentioned or concept discussed in this manuscript.

## Data Availability

Study data are available from the corresponding author upon reasonable request.
